# Supplementary material for: A cell-based scrambling assay reveals the phospholipid headgroup preference of TMEM16F on the plasma membrane
Source: Proc Natl Acad Sci U S A. 2025 Oct 30;122(44):e2516822122. doi: 10.1073/pnas.2516822122 (PMC12595458; doi:10.1073/pnas.2516822122)
Supplement: Supplementary file 1 — Appendix 01 (PDF) [file pnas.2516822122.sapp.pdf]

Supporting Information for

A cell-based scrambling assay reveals phospholipid headgroup preference of TMEM16F on the plasma membrane

Chin Fen Teo[a,b], Sami T. Tuomivaara[c,1], Niek van Hilten[d], David Crottès[a,b,2], Michael Grabe[d], Yuh Nung Jan[a,b], Lily Y. Jan[a,b,*]

[a]Howard Hughes Medical Institute, University of California, San Francisco, CA 94143, USA

[b]Department of Physiology, University of California, San Francisco, CA 94143, USA

[c]Department of Obstetrics, Gynecology, and Reproductive Sciences, Center for Reproductive Sciences, Eli and Edythe Broad Center for Regeneration Medicine and Stem Cell Research, Sandler-Moore Mass Spectrometry Core Facility, University of California, San Francisco, CA 94143, USA

[d]Department of Pharmaceutical Chemistry, Cardiovascular Research Institute, University of California, San Francisco, CA 94143, USA

[1]Current affiliations: Meilahti Proteomics Unit, Department of Biochemistry and Developmental Biology, Faculty of Medicine, Helsinki Institute of Life Science, University of Helsinki, 00014 Helsinki, Finland

[2]Current affiliations: Inserm UMR 1069 N2Cox, Niche, Nutrition, Cancer & Métabolisme Oxydatif, Tours, France

Lily Y. Jan.

E-mail: lily.jan@ucsf.edu

This PDF file includes:

Figs. S1 to S3

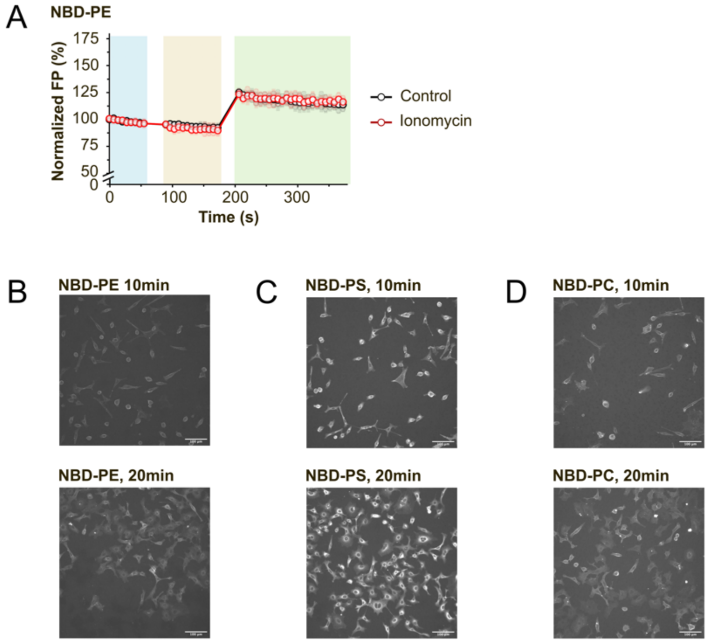

Fig. S1. NBD-PE is prone to both internalization and hydrolysis, hence necessitating shortening of the scrambling duration.

(A) NBD-PE loaded HeLa wild type cells were incubated with ionomycin (*red traces*) or vehicle control (*black traces*) for 90 s (*N*=3, in contrast to 30 s which is the final optimized duration in the manuscript) before Alb addition. Unlike the discernible difference in the Alb-FP between ionomycin and control observed in Fig. 2C and 3C of the manuscript that suggests an increase in NBD-PE on the outer leaflet after 30 s post-stimulation, there is no difference in the ionomycin- and control-treated conditions when the scrambling duration is prolonged to 90 s. The kinetic recording was paused for manual addition of ionomycin as indicated with the white gap between the baseline (*blue shading*) and scrambling (*yellow shading*) stages; and also for manual addition of Alb in as indicated with the white gap before the binding stage (*green shading*). (B to D) The distinct metabolism profiles of each NBD-PL were observed using a widefield epifluorescence Nikon TiE eclipse microscope using a 20x objective (NA 0.45). (B) A549 cells treated with NBD-PE for 10 min (top) display an overall fluorescent signal that indicates plasma membrane localization, while A549 cells treated with NBD-PE for 20 min (bottom) display a weak and punctate fluorescent signal that indicates intracellular membrane localization and hydrolysis. Such a profile is in stark contrast to

that of NBD-PS- (C, also prone to internalization, but not hydrolysis) and NBP-PC- (D, more resistant to both internalization and hydrolysis) loaded A549 cells: Although NBD-PS is also prone to internalization (comparing Panel C top vs bottom), it is more resistant to hydrolysis, reflecting in a much stronger intracellular membrane labeling after 20 min incubation, owing to a more tightly packed intracellular membrane, thus amplifying the environmental sensing nature of the NBD probe. Whereas NBD-PC tends to remain on the plasma membrane during the identical time frame (comparing Panel D top vs bottom). The loading of NBD-PLs was performed in parallel, the images were captured with identical laser intensity settings, and the image displays were also presented using identical parameters for brightness/contrast. The distinct fluorescent profiles of NBD-PLs, in addition to their intrinsic bilayer distributions, necessitate the adjustment of scrambling duration to obtain maximal signal-to-noise in their respective Alb-FPs in the presence or absence of ionomycin stimulation.

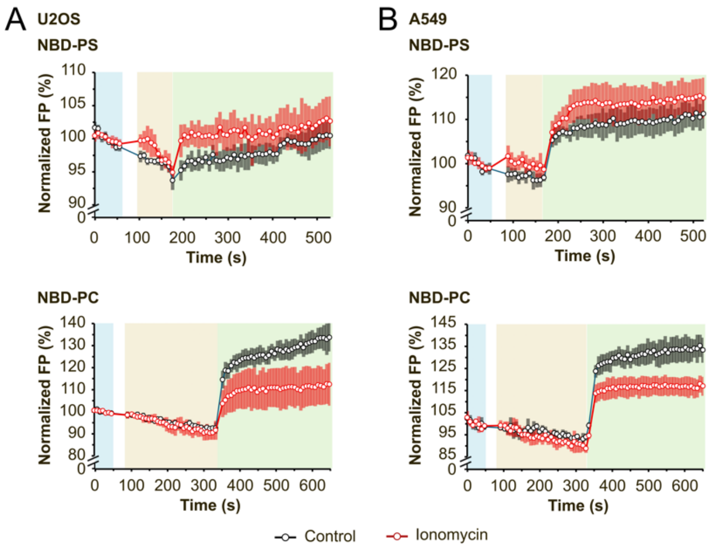

Fig. S2. FP assay also reports PC and PS scrambling in U2OS and A549 cells.

The ability of the FP assay to discern the directionality of PL movement on the plasma membrane was verified using two additional cell lines, (A) U2OS and (B) A549. The data from both cell lines corroborates the findings in HeLa cells: In NBD-PS preloaded cells, the FP signal from scrambling conditions (*red traces*) is higher than in the control (non-scrambling, *black traces*) conditions, and in NBD-PC preloaded cells, the FP signal from scrambling conditions (*red traces*) is lower than in the control (non-scrambling, *black traces*) conditions. For each trace, N = 5. Means (*circles*) ± 1 standard deviation (*shading*) are indicated. The kinetic recording was paused for manual addition of ionomycin as indicated with the white gap between the baseline (*blue shading*) and scrambling (*yellow shading*) stages.

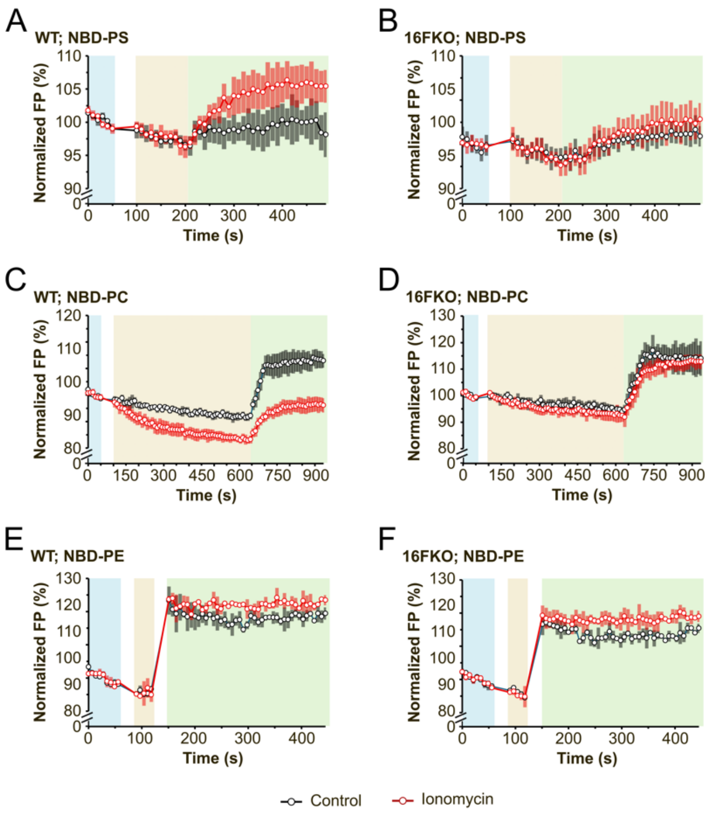

Fig. S3. The full time-course data of PS, PC and PE scrambling in HeLa WT and 16FKO cells. Data related to Fig. 3.

Full time-course data of the kinetic calcium-induced phospholipid scrambling in WT (A, C, E) and 16FKO (B, D, F) HeLa cells. In all cases, the difference between scrambling (red *traces*) and non-scrambling (*black traces*) conditions is narrower in the 16FKO cells compared to the WT cells. For A and B (NBD-PS), $N = 7$, for C and D (NBD-PC), $N = 7$, and for E and F (NBD-PE), $N = 2$. Means (*circles*) $\pm$ 1 standard deviation (*shading*) are indicated. In NBD-PC and NBD-PS preloaded cells, the differences are nearly abolished, indicating that TMEM16F contributes to most of the calcium-activated PC- and PS-scrambling activities in HeLa cells. The insignificant difference between scrambling (red *traces*) and non-scrambling (*black traces*) conditions in NBD-PE preloaded 16FKO cells and WT cells suggests a relatively minor effect on calcium-activated PE scrambling activity in cells lacking TMEM16F. The kinetic recording was paused for manual addition of ionomycin as indicated with the white gap between the baseline (*blue shading*) and scrambling (*yellow shading*) stages. Additionally, the

kinetic recording was also paused for manual addition of Alb in (E and F) as indicated with the white gap before the binding stage (*green shading*).
